# Identification of H_2_O_2_ induced oxidative stress associated microRNAs in HLE-B3 cells and their clinical relevance to the progression of age-related nuclear cataract

**DOI:** 10.1186/s12886-018-0766-6

**Published:** 2018-04-13

**Authors:** Song Wang, Chenjun Guo, Mengsi Yu, Xiaona Ning, Bo Yan, Jing Zhao, Angang Yang, Hong Yan

**Affiliations:** 1 Department of Ophthalmology, Tangdu Hospital, Fourth Military Medical University, 1 Xinsi Road, Xi’an, Shaanxi 710038 People’s Republic of China; 2 Department of Dermatology, Xijing Hospital, Fourth Military Medical University, 169 West Changle Road, Xi’an, Shaanxi 710032 People’s Republic of China; 3 The State Key Laboratory of Cancer Biology, Department of Biochemistry and Molecular Biology, Fourth Military Medical University, 169 West Changle Road, Xi’an, Shaanxi 710032 People’s Republic of China; 4 Chongqing Key Laboratory of Ophthalmology and Chongqing Eye Institute, The First Affiliated Hospital of Chongqing Medical University, 1 Youyi Road, Chongqing, 400016 People’s Republic of China

**Keywords:** Age-related nuclear cataract, Oxidative stress, microRNA, Bioinformatics analysis

## Abstract

**Background:**

This study is aimed to screen out the microRNAs (miRNAs) associated with H_2_O_2_ induced oxidative stress in human lens epithelial B3 (HLE-B3) cell lines and investigate their relations with the progression of age-related nuclear cataract.

**Methods:**

H_2_O_2_ was used to induce oxidative stress in HLE-B3 cells. A genome-wide expression profiling of miRNAs in HLE-B3 cells was performed to select the differentially expressed miRNAs before and after H_2_O_2_ treatment. The selected miRNAs were validated by RT-PCR and fluorescence in situ hybridization (FISH). Clinical specimens were divided into three groups according to the Lens Opacities Classification System III (LOCSIII) and the expression levels of the selected miRNAs were tested by RT-PCR in the three groups. Bioinformatics analyses were applied to predict the target genes of the miRNA hits and construct the miRNA regulatory network. The expression level of MAPK14 was analyzed by Western blot.

**Results:**

The H_2_O_2_ induced oxidative stress model of HLE-B3 cells was established. Nineteen upregulated and 30 downregulated miRNAs were identified as differentially expressed miRNAs. Seven of the total 49 were validated in the cell model. RT-PCR of the clinical samples showed that the expression levels of miR-34a-5p, miR-630 and miR-335-3p were closely related with the severity of nuclear opacity. The images taken from FISH confirmed the results of RT-PCR. There were 172 target genes of the three miRNAs clustered in the category of response to stress. The regulatory network demonstrated that 23 target genes were co-regulated by multiple miRNAs. MAPK14 was the target gene of three miRNAs and the result were verified by Western blot.

**Conclusion:**

Up-regulation of miR-34a-5p and miR-630 and down-regulation of miR-335-3p are related with the progression of age-related nuclear cataract and the underlying mechanism awaits further functional research to reveal.

## Background

Human lenses are transparent in young people, but changes occur as the body ages. These changes include the development of a hard, compact nucleus, local opacity, and, finally, the development of a pathological cataract [1]. By far, many factors such as diabetes mellitus, ultraviolet, systemic drugs and congenital diseases are known to be related to cataract formation. Among these factors, oxidative stress with the generation of reactive oxygen species (ROS) is thought to be a major predisposing factor in age-related cataracts [2]. Substantial data suggest that, with increasing age, the lens nucleus becomes more susceptible to oxidation and less able to repair oxidative damage [3, 4].

MicroRNAs (miRNAs) are evolutionarily well-conserved, small non-coding transcripts. It plays an important role in the post-transcriptional regulation of target mRNA via mRNA degradation or translational repression through binding with 3′-untranslated regions (UTRs) of target genes [5–7]. Accumulating evidences demonstrated that miRNAs play a critical role in multiple pathological processes of mammalian lens [8–10]. A clinical research revealed that the expression profile of miRNAs in cataractous lenses is different from transparent lenses [1]. And further mechanistic study showed that miR-26, miR-30a and miR-211 involved in the formation of cataract through targeting certain mRNAs [11–13]. However, there has no record of a systemic screening for oxidative stress associated miRNAs in human lens epithelial cells (HLECs).

In the present study, we used hydrogen peroxide to induce oxidative damage in human lens epithelium B3 (HLE-B3) cells and monitored the status of cell viability and apoptosis. Subsequently, the miRNA transcriptome profiles of control and oxidized cells were determined by microarray and the differentially expressed miRNAs were validated by RT-PCR. The central epithelium of cataractous human lenses was divided into three groups according to the Lens Opacities Classification System III (LOCSIII) [14] and the expression levels of the distinct miRNAs were verified in these specimens. Finally, bioinformatics analysis was used to find novel targets of cataractogenesis.

## Methods

### Cell culture and treatment

HLE-B3 cells purchased from the American Type Culture Collection (ATCC, Manassas, VA, USA) were grown as a monolayer in DMEM supplemented with 20% heat-inactivated fetal bovine serum (FBS) at 37 °C in a humidified atmosphere of 5% CO_2_ and 21% O_2_. Twenty-four h before the day of the experiment, cells were switched to hypoxic conditions (1% O_2_ to mock physiological environment [15]). At 85–90% confluence, the cells were treated with the indicated concentration of H_2_O_2_ for 24 h.

### Tissue extraction and grouping

Forty five lens epithelium samples, collected from 45 patients (patient age range was 57–86 years, free of other ocular diseases), were obtained by intact continuous curvilinear capsulorhexis. Cataract type and severity were graded in accordance with the LOCSIII. All LOCSIII scorings among subjects were carried out and consisted up to at least three ophthalmologists.

The research population was divided into three groups according to the grading of nuclear opacity (0 < *N* ≤ 2, 2 < *N* ≤ 4, 4 < *N* ≤ 6). There were no statistically significant differences between each group with respect to age or sex of the patient (*P* > 0.05, Independent Sample t-test). This study was performed according to the tenets of the Declaration of Helsinki for Research Involving Human Tissue. Verbal consent was obtained from each patient following an explanation of the surgery procedure and the purpose of this research. The Moral and Ethical Committee of the Fourth Military Medical University approved the verbal consent.

### Cell viability

The MTT assay was used to monitor the viability of HLE-B3 cells. Cells were plated at a density of 5 × 10^3^ cells/well in 96-well microplates. After incubation, cells were treated with different concentrations of H_2_O_2_ for a different duration time. Then cells were incubated with 20 μl of MTT solution (0.5 mg/ml) for 4 h at 37 °C. The incubation was stopped by removing the culture medium and 200 μl DMSO was added to solubilize formazan. The absorbance was measured at 490 nm by a microplate reader (Bio-Rad, West Berkeley, CA).

### Detection of cell apoptosis by flow cytometry

Cells were incubated in a six-well plate at a density of 5 × 10^5^ cells per well. After treatment, the cells were washed twice with PBS and harvested by trypsin digestion. Cells were collected and centrifuged at 500 rpm for 5 min, the supernatant was discarded, and 5 μl of Annexin V-FITC and 10 μl of propidium iodide (PI) were added to the cell pellet followed by 15 min incubation in the dark at room temperature. Samples were analyzed by flow cytometry within 60 min of processing.

### Hoechst staining

Cells were stained with 10 μg/ml Hoechst 33,258 in the dark at room temperature for 5 mins, after which the cells were washed twice with PBS. The nuclear morphology of stained cells was examined using a fluorescence microscope with an excitation of 350 nm and emission of 460 nm. Nonspecific fluorescence values were subtracted from the experimental fluorescence values. At least 100 cells in three different fields were counted, and the data are presented as the percentage of viable cells out of the total number of cells.

### RNA isolation and real-time PCR

Total RNA from cells and tissues were isolated using TRIzol Reagent (Invitrogen, Carlsbad, CA, USA) according to manufacturer’s instructions. RNA content was measured using a Nanodrop-2000 (Thermo Fisher Scientific, Waltham, MA, USA). First strand cDNA was synthesized from the total RNA of the HLE-B3 cells and tissue samples using the miScript Reverse Transcription Kit (Qiagen, Germany) in accordance with the manufacturer’s recommended protocol. The quantitative real-time PCR (qRT-PCR) was conducted using the SYBR Green dye (TaKaRa, Japan). Real-time PCR was performed in triplicate on CFX96 Real Time PCR Detection System (Bio-Rad, West Berkeley, CA). The 2ˆ-ΔΔCT method was used to determine the relative gene expression, and mature miRNA were normalized to U6-snRNA.

### Microarray analysis

For microRNA expression analysis, total RNA, including microRNA, was isolated from treated and control groups of HLE-B3 cells according to the manufacturer’s instructions and analyzed using Affymetrix GeneChip miRNA 2.0 arrays (Affymetrix, Santa Clara, CA, USA) containing 4560 probe sets for human small RNAs. All of the steps of the procedure were performed according to the standardized protocol for Affymetrix miRNA 2.0 arrays. The intensity values for microRNA transcripts were calculated using Affymetrix GeneChip Command Console 3.2. The quality control for the microarray was performed with the Affymetrix miRNA QC Tool.

### Western blot analysis

Cells were harvested, rinsed in PBS, and lysed in RIPA buffer containing 5% PMSF for 1 h on ice. After the mixture was centrifuged at 12,000×g for 10 min at 4 °C, insoluble materials were removed. Identical amount (50 μg of protein) of cell lysates were boiled for 5 min, size fractionated by SDS-PAGE, and electrophorectically transfected on to PVDF membranes. After being incubated with blocking solution including 5% powered milk in TBST buffer (10 mM Tris–HCl, 150 mM NaCl, and 0.1% Tween-20) for 1 h at room temperature, the membranes were immunoblotted with primary antibodies (Cell Signaling Technology, USA. Catalogue 9211, 9212). Primary antibodies were identified using HRP-conjugated secondary antibody at a 1:10,000 dilution and visualized by the ECL detection system.

### Fluorescence in situ hybridization (FISH)

Specific probes of miR-34a-5p, miR-630 and miR-335-3p were used in FISH and the sequences are 5’-ACAACCAGCTAAGACACTGCCA-3′ for miR-34a-5p, 5’-ACCTTCCCTGGTACTGAATACT-3′ for miR-630 and 5’-GGTCAGGAGCAATAATGAAAAA-3′ for miR-335-3p. In brief, HLEB-B3 cells were treated with H_2_O_2_ for 24 h. 5′ cy3-labelled probes were specific to the miRNAs. Nuclei were stained by 4,6-diamidino-2-phenylindole (DAPI). All the procedures were conducted according to the manufactory’s instruction (Genepharma, Shanghai, China). All images were observed using fluorescent microscopy (Nikon, Eclipse CI, Tokyo, Japan).

### Bioinformatics analysis of the differentially expressed microRNAs

Target genes of the distinct miRNAs were determined by the union of miRNA target predictions from TargetScan 7.1 [7] (http://www.targetscan.org) and miRanda [16] (http://www.microrna.org). The picked genes were further analyzed according to the PANTHER classification system [17] (http://www.pantherdb.org). Finally, visualization of the miRNA-mRNA regulatory network was achieved by Cytoscape [18].

### Statistical analysis

Statistical analyses and data imaging were performed using GraphPad Prism version 5.00 (GraphPad Inc., La Jolla, CA, USA). Quantitative data was presented as means±SD from at least three separate experiments. Two-tailed Student’s *t*-test was used to evaluate experiments with two experimental groups. The results were considered statistically significant when *P < 0.05; **P < 0.01; ***P < 0.001.

## Results

### H_2_O_2_ treatment decreased the viability of HLE-B3 cells

**Fig. 1:**
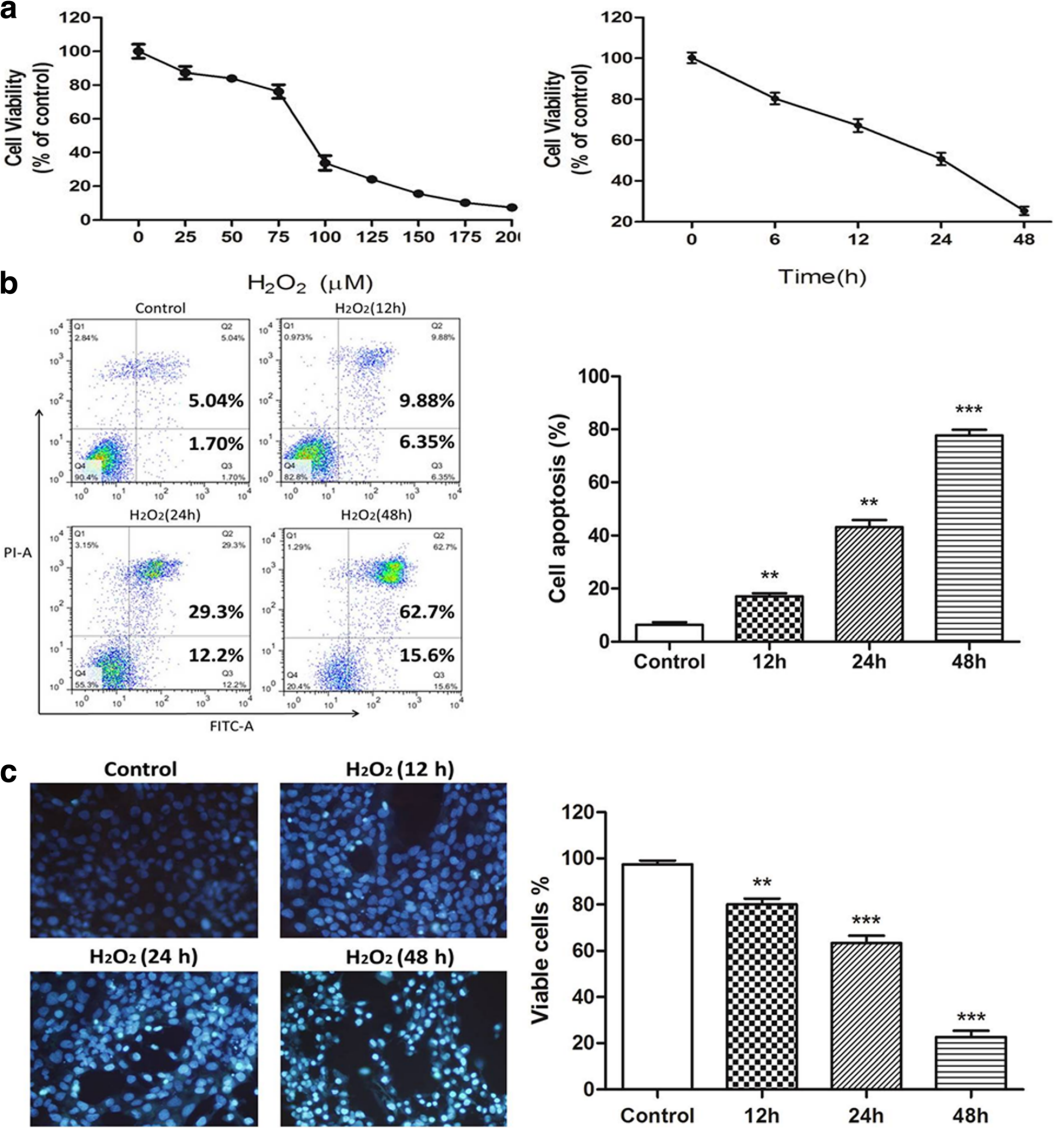
Establishment of H_2_O_2_ induced oxidative stress model in HLE-B3 cells. **a** The viability of HLE-B3 cells decreased after H_2_O_2_ treatment in both dose-dependent and time-dependent manner. **b** Cell apoptosis of HLE-B3 cells after H_2_O_2_ exposure for 12, 24 and 48 h was determined by flow cytometry with Annexin-V and PI staining. **c** Cell nucleus apoptosis of HLE-B3 cells after H_2_O_2_ exposure for 12, 24 and 48 h was determined by Hoechst 33,258 staining. Results are presented as mean ± SD by *t*-test. (*n* = 3) **P < 0.01, ***P < 0.001

In the current study, we used H_2_O_2_ to generate excessive ROS, which can permeate cellular membranes and enter into the cells to cause oxidative damage. The viability of HLE-B3 cells exposed to various concentrations of H_2_O_2_ after 24 h incubation was investigated by MTT assay. Generally, the toxicity of H_2_O_2_ increased in a dose-dependent manner as shown in Fig. 1a. From 0 μM to 75 μM, cell viability decreased gently. However, from 75 μM to 100 μM, cell viability dropped drastically from 76.22 ± 2.64% to 33.76 ± 2.20%. Meanwhile we found that, at the concentration of 75 μM, H_2_O_2_ decreased the viability of HLE-B3 cells in a time-dependent manner (Fig. 1a). Therefore, in order to achieve the balance between oxidative damage and cell survival, the treatment of 75 μM H_2_O_2_ for 24 h was chosen to induce oxidative stress on HLE-B3 cells for further research.

### H_2_O_2_ treatment induced apoptosis in HLE-B3 cells

Flow cytometry was used to quantify the rate of apoptosis using double staining of Annexin V-FITC and PI. As the results shown in Fig. 1b, HLE-B3 cells treated with 75 μM H_2_O_2_ showed significant apoptosis, besides, the apoptosis rate increased in a time-dependent manner. To validate the flow cytometric data for H_2_O_2_-induced apoptotic cell death, Hoechst 33,258 staining was used to detect apoptotic cell nucleus. Normal nuclear morphology (round blue nuclei) was observed in the H_2_O_2_-free control group (Fig. 1c). However, chromatin condensation and strong fluorescent spots were observed in HLE-B3 cells treated with 75 μM H_2_O_2_. The percentage of Hoechst-positive cells correlated closely with the percentage of Annexin V-positive cells in Fig. 1b.

### Microarray screening for microRNAs associated with H_2_O_2_ induced oxidative stress in HLE-B3 cells

**Fig. 2:**
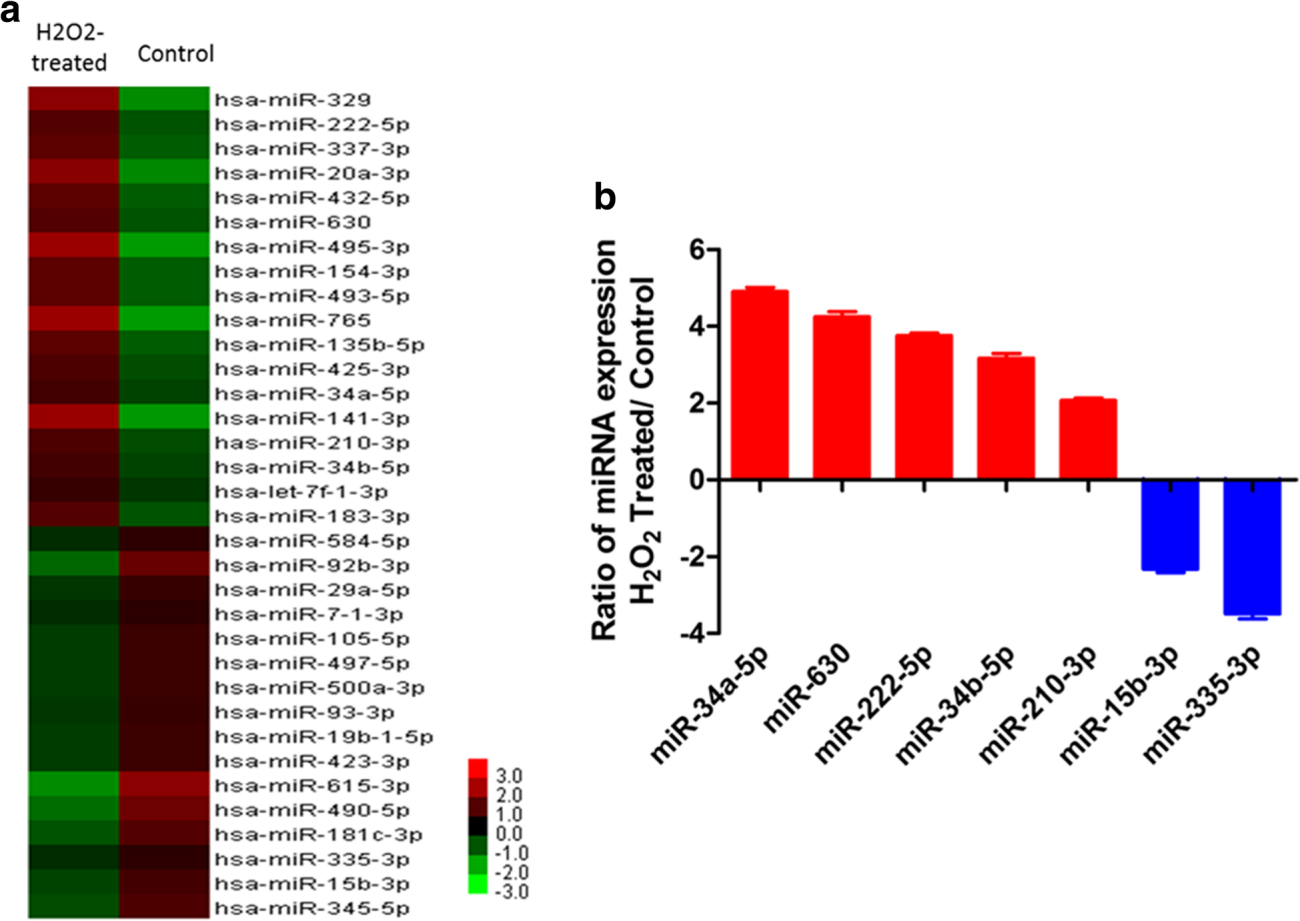
Microarray screening for differentially expressed miRNAs which were induced by oxidative stress in HLE-B3 cells. **a** Heat map of miRNAs that are differentially expressed between the H_2_O_2_-treated group and the control group. **b** Quantitative real-time RT-PCR validation of five up-regulated and two down-regulated miRNAs (mean ± SD, *n* = 3)

Microarray analysis was used to characterize the H_2_O_2_ induced miRNAs. Microarray data revealed that after H_2_O_2_ treatment, 19 miRNAs were upregulated 2-fold relative to the control group; 30 miRNAs were downregulated 2-fold (Fig. 2a). Ultimately, according to the miRNAs’ fold change and expression level, five up-regulated miRNAs (miR-630, miR-222-5p, miR-210-3p, miR-34a-5p and miR-34b-5p) and two down-regulated miRNAs (miR-335-3p and miR-15b-3p) were chosen for microarray validation by RT-PCR (Table 1). The result of the PCR analysis confirmed the differentially expressed miRNAs selected by microarray (Fig. 2b).

**Table 1:** Fold change of the seven selected miRNAs and their forward primer sequences used for RT-PCR

miRNA Name	Fold Change	Forward Primer for RT-PCR
miR-630	4.14	GCGAGTATTCTGTACCAGGGAAGGT
miR-222-5p	3.84	CGCTCAGTAGCCAGTGTAGATCCT
miR-210-3p	3.23	CTGTGCGTGTGACAGCGG
miR-34a-5p	2.59	CTGGCAGTGTCTTAGCTGGTTGT
miR-34b-5p	2.44	GCGTAGGCAGTGTCATTAGCTGATTG
miR-335-3p	0.45	CGGCGTTTTTCATTATTGCTCCTGACC
miR-15b-3p	0.33	CGGGCGAATCATTATTTGCTGCTCTA

### The association between the oxidation-induced miRNAs and nuclear opacity

**Fig. 3:**
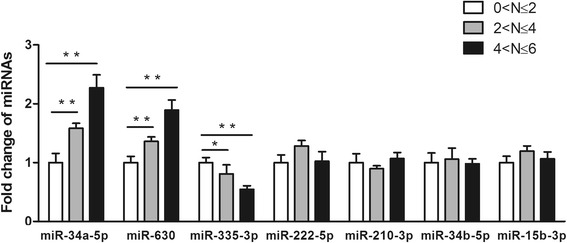
Relevance of expression levels of the seven miRNAs to the severity of lens nuclear opacity. Forty five samples were divided into three groups according to their grading of nuclear opacity. Each group contained 15 samples. The expression level of each miRNA in 0 < *N* ≤ 2 group was defined as 1. Up-regulation of miR-34a-5p and miR-630 are closely related to a higher severity score of nuclear cataract, while down-regulation of miR-335-3p is associated with the increase of nuclear opacity (mean ± SD, *n* = 3). *P < 0.05, **P < 0.01

To identify the connections between the grading of nuclear opacity and expression levels of miRNAs, Pearson correlation coefficient was introduced (Fig. 3). For miR-34a-5p, miR-630 and miR-335-3p, close relations (*R* = 0.691, 0.617, − 0.594) between LOCSIII grading and their expression levels were found and they were statistically significant (*P* < 0.001). However, for miR-222-5p, miR-210-3p, miR-34b-5p and miR-15b-3p, the relations were moderate (*R* = 0.436, 0.428, 0.398, 0.489) and statistically insignificant (*P* > 0.05).

Then we compared the expression levels of miR-34a-5p, miR-630 and miR-335-3p in each subgroup (Fig. 3). In miR-34a-5p and miR-630, higher scores of nuclear opacity associated with greater levels of both miRNAs (0 < *N* ≤ 2 as control, *P* < 0.01). Meanwhile, in miR-335-3p, higher grading correlated with lower levels of miR-335-3p (0 < N ≤ 2 as control, *P* < 0.05).

### Validation of the levels of miR-34a-5p, miR-630 and miR-335-3p by FISH

**Fig. 4:**
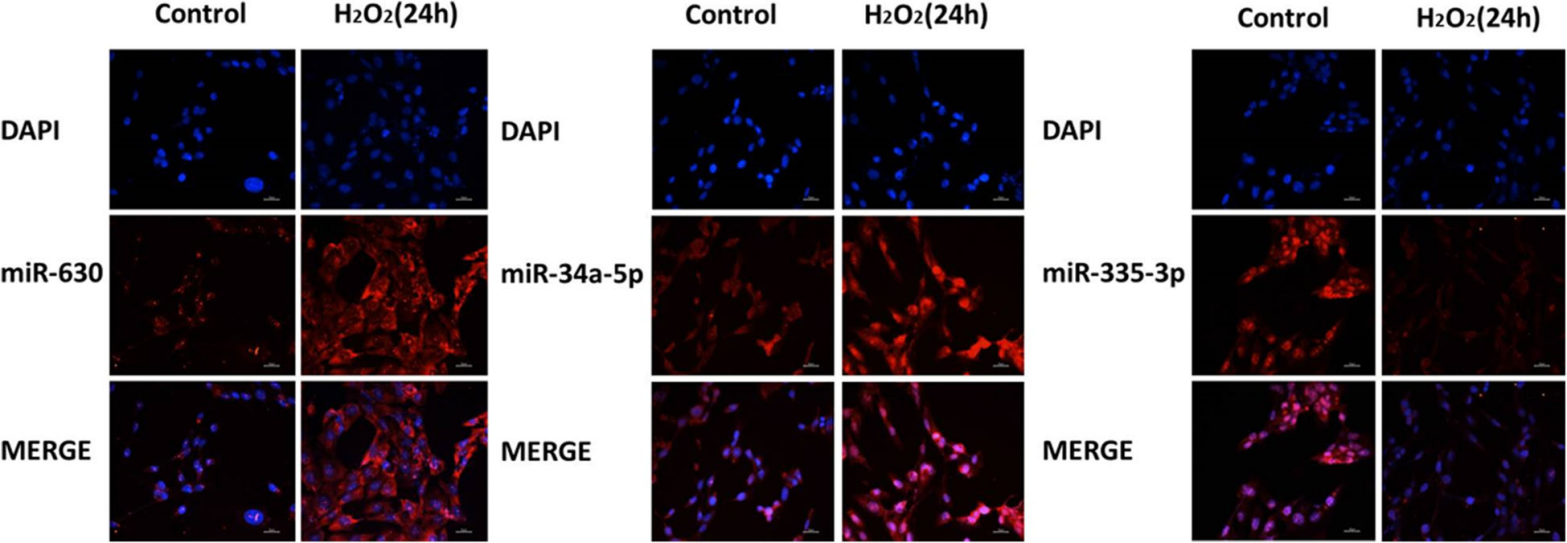
The expression of three key miRNAs in HLE-B3 cells were assessed by FISH. (400×, scale bar is 25 μm)

After evaluating the PCR results of HLE-B3 cells and clinical samples, we chose miR-34a-5p, miR-630 and miR-335-3p as the key miRNAs in our research. In order to further validate the PCR tests, FISH was applied and images were taken to estimate the difference visually. As Fig. 4 showed that, after 24 h of H_2_O_2_ treatment, the expression levels of miR-630 and miR-34a-5p were elevated while miR-335-3p was down-regulated. The results were in accordance with the microarray and PCR tests.

### Identification of target genes and analysis of the miRNA/target gene network

**Fig. 5:**
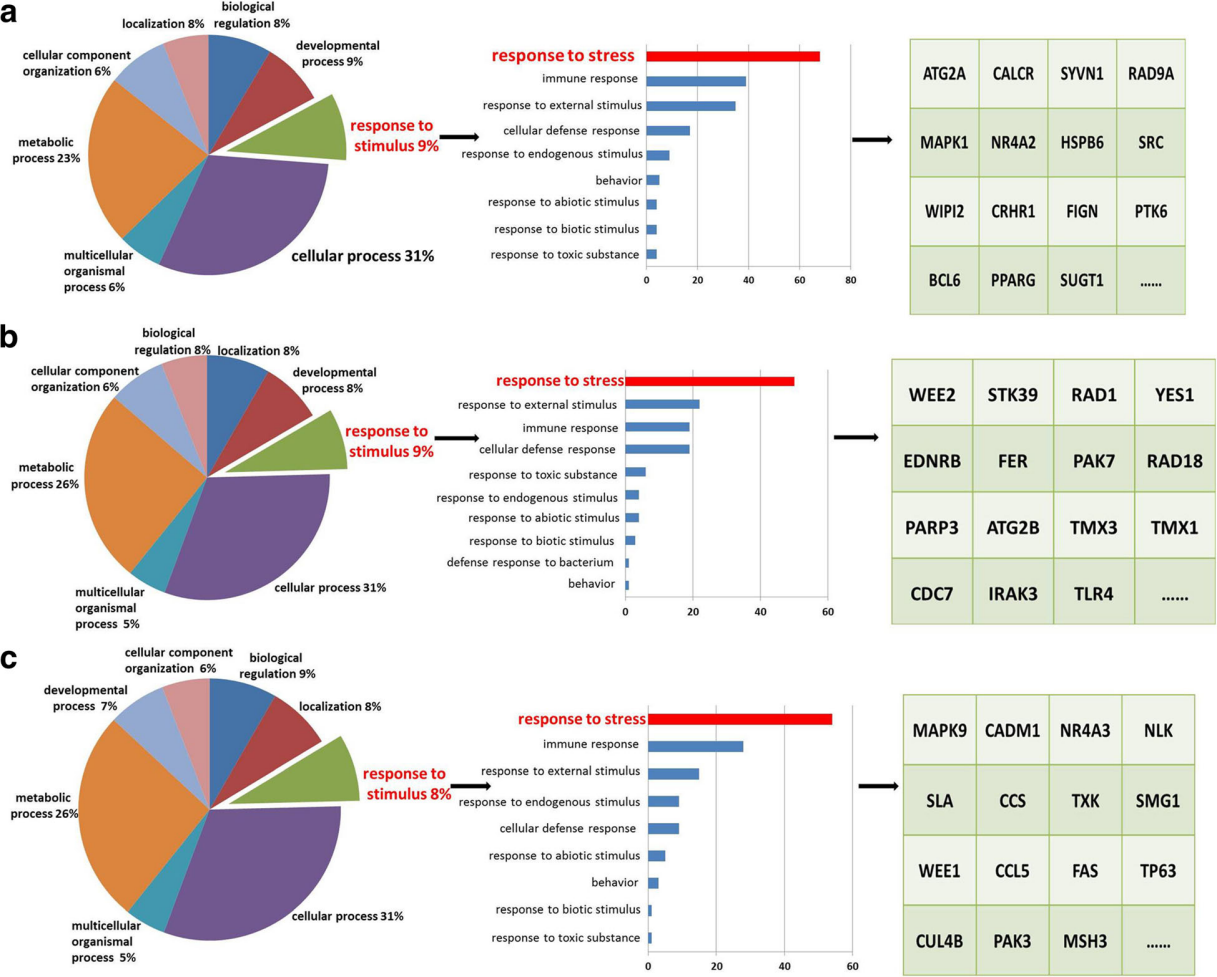
Target classification of the three selected miRNAs. **a** Target selection of miR-34a-5p. Sixty-eight target genes of miR-34a-5p were clustered in the process of response to stress. **b** Target selection of miR-630. Fifty target genes of miR-630 were clustered in the process of response to stress. **c** Target selection of miR-335-3p. Fifty-four target genes of miR-335-3p were clustered in the process of response to stress

**Fig. 6:**
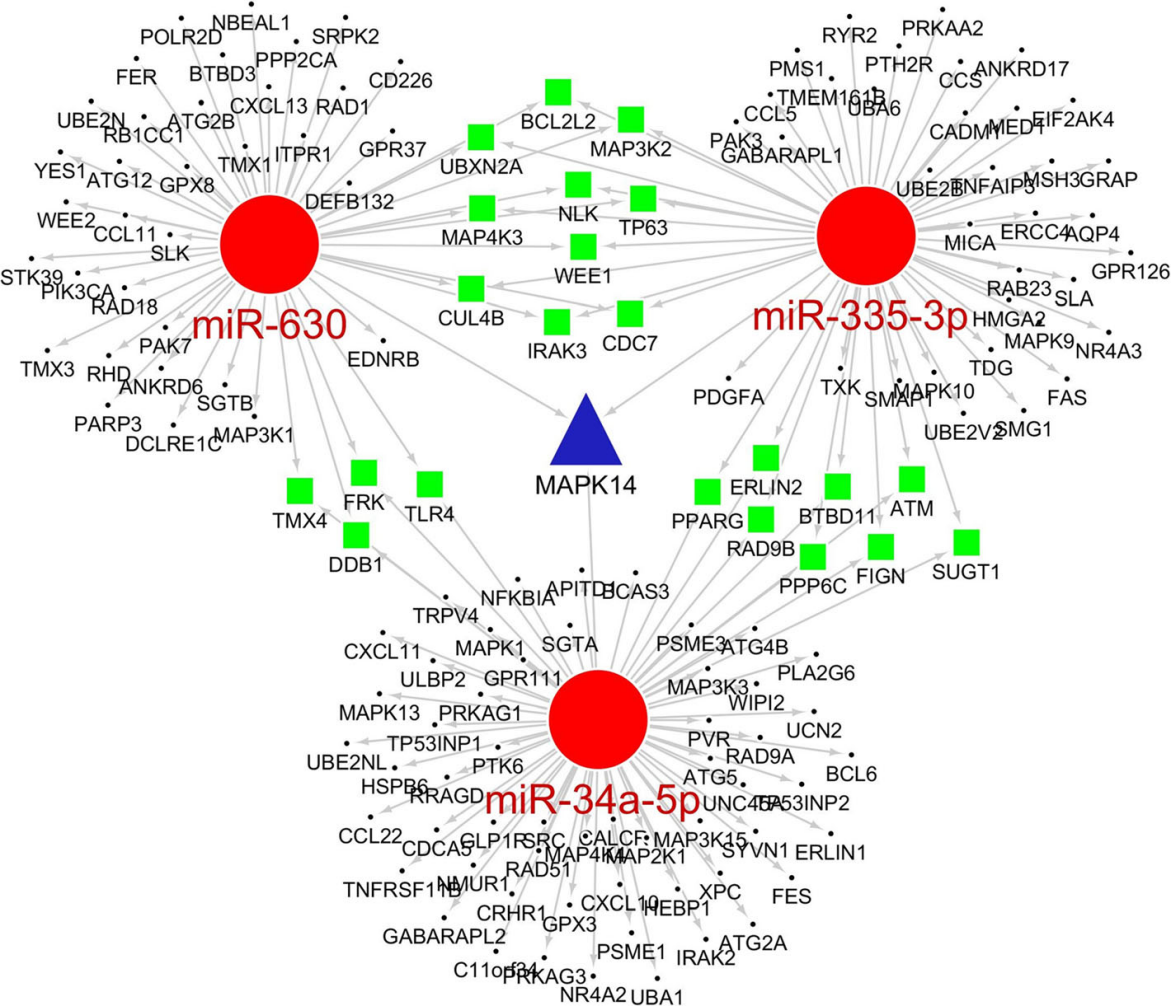
Network of the miRNAs and their target genes in the process of response to stress. Red circles, miRNAs; black dots, target genes regulated by one miRNA; green squares, target genes regulated by two miRNAs; blue triangle, target gene regulated by three miRNAs

**Fig. 7:**
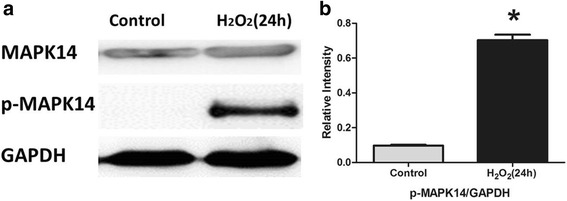
Western blot analysis of MAPK14 and p-MAPK14 in H_2_O_2_ treated HLE-B3 cells. **a** The expression level of MAPK14 remained the same after 24 h H_2_O_2_ treatment while p-MAPK14 increased significantly. **b** Quantitative analysis of the relative intensity of protein levels in HLE-B3 cells. (*n* = 3,*P < 0.05)

According to Targetscan and miRanda, there were 1532, 1323 and 1275 genes predicted to be the target genes of miR-34a-5p, miR-630 and miR-335-3p. Predictions from the algorithms were submitted to the PANTHER classification system. First, genes were distributed to different biological processes. Then we picked the process of response to stress, which is a subcategory of response to stimulus, as our target process. The picked genes from the algorithms were further analyzed according to the PANTHER classification system, in which 68, 50 and 54 genes were clustered in the response to stress process (Fig. 5a-c). The network of miRNA/targets was constructed using Cytoscape (Fig. 6). Furthermore, we screened out 23 genes which were co-regulated by miRNAs and MAPK14 was the target gene of all three miRNAs (Tables 2 and 3). Western blot analysis showed that the level of phospho-MAPK14 (p-MAPK14) increased when cells were exposed to H_2_O_2_ for 24 h while the level of MAPK14 remained the same (Fig. 7).

**Table 2:** Target genes clustered in response to stress by PANTHER classification system

MiRNA	Clustered target genes
miR-34a-5p	ATG2A, CALCR, SYVN1, RAD9A, TP53INP2, MAPK14, MAPK1, NR4A2, BTBD11, HSPB6, NMUR1, WIPI2, CRHR1, MAP3K15, FIGN, PTK6, TRPV4, BCL6, RRAGD, ATG4B, MAP2K1, TNFRSF11B, PPARG, CCL22, GABARAPL2, ERLIN2, PVR, SUGT1, SRC, GPR111, NFKBIA, MAPK13, XPC, GLP1R, FRK, C11orf34, TLR4, TP53INP1, SGTA, ERLIN1, UBA1, TMX4, UCN2, UNC45A, APITD1, CDCA5, HEBP1, ATM, PSME1, CXCL11, ATG5, MAP3K3, RAD9B, PSME3, DDB1, ULBP2, FES, BCAS3, PRKAG1, PRKAG3, PLA2G6, PPP6C, RAD51, IRAK2, UBE2NL, MAP4K4, GPX3,CXCL10
miR-630	WEE2, MAP3K2, MAP4K3, STK39, DEFB132, MAPK14, ANKRD6, RAD1, ATG12, SGTB, MAP3K1, YES1, RB1CC1, BCL2L2, EDNRB, WEE1, FER, PAK7, DCLRE1C,UBXN2A, RAD18, PARP3, SLK, ATG2B, TMX3, BTBD3, TMX1, GPR37, CDC7, PPP2CA, IRAK3, TP63, RHD, FRK, CXCL13, POLR2D, GPX8, ITPR1, TLR4, SLK, UBE2N, SRPK2, CUL4B, TMX4, NBEAL1, CD226, CCL11, PIK3CA, DDB1, NLK
miR-335-3p	MAPK9, CADM1, MAP3K2, PRKAA2, MAP4K3, NR4A3, GRAP, MAPK14, EIF2AK4, BTBD11, NLK, ANKRD17, SLA, CCS, UBE2B, FIGN, PPP6C, TXK, ERCC4, PDGFA, GPR126, BCL2L2, SMG1, WEE1, CCL5, UBXN2A, PPARG, FAS, ERLIN2, RYR2, CDC7, SUGT1, MED1, AQP4, TDG, PTH2R, IRAK3, PMS1, UBA6, MICA, TP63, HMGA2, TNFAIP3, CUL4B, SMAP1, PAK3, GABARAPL1, UBE2V2, ATM, MSH3, MAPK10, RAD9B, TMEM161B,RAB23

**Table 3:** Genes targeted by at least two miRNAs in the cluster of response to stress

Co-regulated miRNAs	Target genes
miR-34a-5p/miR-630	DDB1, TMX4, FRK, TLR4
miR-34a-5p/miR-335-3p	PPARG, ERLIN2, RAD9B, BTBD11, PPP6C, ATM, FIGN, SUGT1
miR-630/miR-335-3p	UBXN2A, BCL2L2, MAP3K2, MAP4K3, NLK, TP63, WEE1, CUL4B, IRAK3, CDC7
miR-34a-5p/miR-630/miR-335-3p	MAPK14

## Discussion

Cataract is the leading cause of blindness worldwide and age-related cataract accounts for the largest percentage [19]. There are three main types of age-related cataract: nuclear, cortical or posterior subcapsular cataracts (PSC). Among the three different types, nuclear cataracts predominate in Chinese population [20]. Researchers and ophthalmologists found out multiple risk factors of nuclear cataracts, including malnutrition, smoking, larger lens and family history. Notably, oxidative stress is considered the major cause of nuclear cataracts [2].

A series of studies focused on hyperbaric oxygen therapy discovered that the treatment, whether long-term or short-term, could lead to a myopic shift, then incipient or full-blown nuclear cataracts [21–23]. These findings provide a direct link between excessive oxygen exposure and nuclear cataract. Further evidence is that patients undergoing vitrectomy had significantly higher rates of nuclear cataract formation (60–95%) within 2 years after the surgery [24–27]. Research revealed that oxygen levels near the lens increased markedly during vitrectomy and remained significantly elevated for months afterward [28]. The hypothesis is that vitrectomy leads to cataract formation by increasing the exposure of the lens to oxygen and Lou’s research explained in detail about the mechanism of protein aggregation and cataract formation caused by oxidation overload [29]. In the present study, in order to simulate the physiological environment of lens, which is in a natural state of hypoxia [30], we switched the HLE-B3 cells into the 1% O_2_ incubator 24 h before the H_2_O_2_ treatment.

The miRNAs have recently emerged as a prominent class of gene regulators. Although miRNAs have been identified as key regulators of multiple pathways involved in cataract formation and development, there is no systemic screening for oxidative stress and cell apoptosis associated miRNAs in HLECs. In the current study, after the induction of cell apoptosis by H_2_O_2_, the authors used microarray to identify the crucial miRNAs. Among the seven selected miRNAs, which were validated in HLE-B3 cells by RT-PCR, three of them were proven to be correlated with age-related nuclear cataract and they are miR-34a-5p, miR-630 and miR-335-3p.

MiR-34a-5p is one of the most explored miRNAs in oxidative stress and cell senescence [31]. Bai et al. found that miR-34a-5p suppressed mitochondrial anti-oxidative enzymes with a concomitant increase in intracellular ROS level [32]. Ito et al. found that miR-34a increased with age in endothelial cells in senescent human umbilical cord vein endothelial cells and in the hearts and spleens of older mice [31]. The regulation of miR-34a-5p/SIRT 1 pathway was investigated in multiple disease and aging models for its effect on aging and oxidative damage [33–35]. In the present research, our data demonstrated that the expression level of miR-34a-5p in the oxidized HLE-B3 cells is 2.59 fold higher compared to the control group. In the clinical specimens, the miRNA’s abundance increases as the LOCSIII grading climbs. Among the seven selected miRNAs, it is ranked NO.1 in not only the Pearson correlation coefficient but also the ratio of fold change. Our findings are in accordance with Chien’s research [36].

Mir-630 has been studied in oncology. Researchers focused on the regulatory effect of miR-630 on epithelial-to-mesenchymal transition (EMT). Through targeting FoxM1 [37] and Slug [38], overexpression of miR-630 is capable of suppressing EMT in various carcinomas. However, there is no report concerning about the correlation of miR-630 with cataract. In our study, the level of miR-630 in HLECs elevated drastically at the presence of H_2_O_2_ (4.14 fold) and this trend remained the same in the cataractous human lenses. Further mechanistic study is needed to confirm the involvement of miR-630 in cataractogenesis, but we can speculate that miR-630 may play an important role in the formation of PSC and posterior capsular opacification (PCO) for its influence in EMT.

Opinions about the role of miR-335-3p in tumor progression are controversial. Some believed that miR-335-3p is an oncogene for its effect on induction of multidrug resistance [39] and cancer-associated fibroblasts [40], while others viewed miR-335-3p as a tumor suppressor [41, 42]. This difference could be explained by the diversity of miRNA’s function. Although the function of miR-335-3p in cataract formation is unclear, the results of our experiment, for the first time, offered evidence that down-regulation of miR-335-3p may be the cause or effect of age-related nuclear cataract.

According to the results of bioinformatics analysis, there were 23 genes co-regulated by the three miRNAs in the process of response to stress. Most of all, we found that MAPK14 was the target gene of all three miRNAs and the Western blot analysis indicated that oxidative stress could induce the phosphorylation and activation of MAPK14. Since it was co-regulated by three miRNAs, the specific mechanism needs further research. Mitogen-activated protein kinase 14 (MAPK14) is a member of the MAP kinase family, which is activated by various environmental stresses and proinflammatory cytokines [43, 44]. Although there is no report considering the connection between MAPK14 and cataract, our finding suggests the possibility of MAPK14’s involvement in cataract formation via its response to oxidative stress.

## Conclusions

In conclusion, our research is the first to conduct a microarray screening for oxidative stress and cell apoptosis associated miRNAs in HLECs. The selected miRNAs were further validated by clinical samples from age-related nuclear cataract patients, which suggest that miR-34a-5p, miR-630 and miR-335-3p might be potential regulators of cataract formation. Among them, miR-630 and miR-335-3p are first reported in the field of cataract research. Further mechanism research is needed to identify these miRNAs’ target genes and functions and these miRNAs may serve as molecular targets for the diagnosis and treatment of age-related cataract.

## Abbreviations

EMT: Epithelial-to-mesenchymal transition
FISH: Fluorescence in situ hybridization
HLEs: Human lens epithelial cells
LOCSIII: Lens Opacities Classification System III
MAP K14: Mitogen-activated protein kinase 14
miRNA: MicroRNA.
PCO: Posterior capsular opacification.
PI: Propidium iodide.
PSC: Posterior subcapsular cataracts.
qRT-PCR: Quantitative real-time PCR.
ROS: Reactive oxygen species.
UTRs: Untranslated regions.
